# Understanding the effectiveness and design of parent-oriented mobile health interventions: a systematic review and narrative synthesis

**DOI:** 10.1186/s12887-025-05656-y

**Published:** 2025-05-10

**Authors:** Alicia Kilfoy, Isabella Zaffino, Enoch McAtee, Prabdeep Panesar, Kristin Cleverley, Quynh Pham, Charlene H. Chu, Lindsay Jibb

**Affiliations:** 1 Lawrence Bloomberg Faculty of Nursing, University of Toronto, 155 College St, Toronto, Canada; 2 Division of Hematology/Oncology, The Hospital for Sick Children, 170 Elizabeth St, Toronto, Canada; 3 Child Health Evaluative Sciences, The Hospital for Sick Children, 676 Bay St, Toronto, Canada; 4 Faculty of Health Sciences, McMaster University, 1280 Main St W, Hamilton, Canada; 5 Centre for Addiction and Mental Health, 479 Spadina Ave, Toronto, Canada; 6 Institute of Health Policy, Management and Evaluation, Dalla Lana School of Public Health, University of Toronto, 155 College St, Toronto, Canada; 7 Centre for Digital Therapeutics, University Health Network, 190 Elizabeth St, Toronto, Canada; 8 KITE-Toronto Rehabilitation, University Health Network, 550 University Avenue, Toronto, Canada

**Keywords:** Parents, Caregivers, Mobile Health, Interventions, Acute and Chronic conditions, Pediatrics

## Abstract

**Background:**

Parents of children with a health condition experience high levels of distress which can have long-term impact on the child and parent. Dyadic interventions have the potential to decrease this distress, however several barriers to access including time constraints have been reported. Mobile health (mHealth) interventions can address several of these barriers.

**Goal:**

The goal of this systematic review was to review and synthesize the literature examining the effects of parent-oriented mHealth interventions and their content and design.

**Methods:**

We searched PubMed/MEDLINE, Embase, PsycINFO, CINAHL and Cochrane Central databases from January 2013 to 2023 using a search strategy based on telemedicine and parents/caregivers. Included studies were randomized controlled trials assessing the effect of parent-oriented mHealth interventions on child and parent health. The Cochrane risk-of-bias tool was used to assess for bias in studies. Trial details and design and content features of interventions were extracted. Outcomes were organized using the Van Houtven’s Framework for Informal Caregiver Interventions. Results are presented narratively.

**Results:**

Fifty papers pertaining to 49 unique studies met our inclusion criteria. More than half of the studies scored high-risk for bias. Interventions targeted a wide range of pediatric conditions. Intervention type included texting (*n* = 17) and investigator-developed mobile applications (*n* = 16). Interventions significantly improved parent psychological health and child health outcomes. Key intervention features and design included the use/application of codesign and a theory-driven intervention.

**Conclusion:**

Parent-oriented mHealth interventions identified in this review significantly improved both parent and child health outcomes. Therefore, these interventions have the potential to support parents outside of a clinical setting.

**Supplementary Information:**

The online version contains supplementary material available at 10.1186/s12887-025-05656-y.

## Background

Parents of children with physical or mental health conditions or disabilities are often expected to take on several roles while caring for their child. These complex roles include being a proxy medical-decision maker, advocate, care coordinator, and provider of direct patient care, responsible for medication administration and assistance with activities of daily living [1, 2]. Parents of children with serious or chronic illnesses, such as cystic fibrosis, diabetes, and cancer, experience significantly higher levels of parenting stress compared to parents of healthy children [3]. Studies show that up to 40% of parents of children with chronic conditions report clinically significant stress, with 38% experiencing moderate to severe anxiety and 26% facing moderate to severe depression [4]. This ongoing stress can severely affect parents'overall quality of life, with up to 45% of parents at risk for a decline in health-related quality of life [5].

A parent’s psychological health has been shown to impact their child’s physical and psychological well-being, including levels of anxiety [1, 6, 7]. Further, studies have found a significant positive association between children’s psychological health and overall family relationships including family cohesion and conflict [8]. This connection between parent and child health underscores why family-centered care models have become integral to pediatric medicine [9]. Parents have reported that having access to resources such as emotional support and information related to clinical knowledge and skills is essential for enhancing their caregiving ability [10–13]. Despite this known connection, parents of ill children have reported barriers to accessing supportive interventions. These barriers stem from a variety of issues including a lack of evidence-based interventions [14–16], limited staff knowledge regarding the delivery of psychosocial interventions [17, 18], and the inability to attend in-person support sessions due to child treatment and other family demands [19].

Mobile health interventions (mHealth), including digital applications, texting with clinicians and automated text-based prompts, have the potential to address several known barriers to parent-oriented interventions [20]. This terminology can be traced back to the pioneering work of Istepanian et al. in 2003, [21–23] who first defined it as emerging mobile computing, medical sensor and communication technologies for healthcare. The field expanded in 2007 with the introduction of the first generation of smartphones [21–23]. In 2011, the World Health Organization (WHO) stated that mHealth has the potential to transform health service delivery globally. The development and growth of these interventions were further accelerated during the COVID- 19 pandemic, due to the need for social distancing and lockdowns [21–23]. Together, interest in these interventions has increased, in part due to their ability to provide enhanced access to personalized support, allowing users to receive assistance in real time and in various non-clinical environments in response to changes in health status or behaviors [20, 24].

The development and design of mHealth interventions is a complex process, and the lack of involvement of intervention users (or end-users) such as patients and families can limit intervention effectiveness, integration into practice and sustainability [25–27]. Co-design of mHealth interventions, in which a diverse range of partners participate in the design and development process [25], is one method to address this issue [28]. However, little is known about the extent to which co-design has been used to guide the development of parent mHealth intervention and its impact.

To date, several reviews have explored the effectiveness and design of mHealth interventions in adults with various health conditions, including dementia and frailty, as well as their family caregivers [29–31]. In pediatrics, one review and meta-analysis focused on pediatric-oriented mHealth interventions found that parent involvement in mHealth interventions led to effect sizes larger than those without parental inovlvement [19]. Other reviews in pediatrics have concentrated on specific acute or chronic conditions [32, 33], have not provided important information regarding design and development [34], shown heterogenous effectiveness results [35] or are not recent [36]. Given the lessons that can be learned across different conditions and the continued exponential growth of mHealth, an updated review addressing each of these gaps is necessary.

Our overarching goal was to synthesize the literature examining the effects of parent-oriented mHealth interventions; as well as the content and design of such interventions. Our specific objectives were to describe: (1) the impact of parent-oriented mHealth interventions on parent health outcomes compared to a control group; (2) the impact of parent-oriented mHealth interventions on child health outcomes compared to a control group; (3) the design, content, and functionality of the identified parent-oriented mHealth interventions; and (4) evaluate the quality of these studies.

## Materials and methods

### Study design, literature search and study selection

A systematic review was conducted. Our reporting is in accordance with the Preferred Reporting Items for Systematic Reviews and Meta-Analyses (PRISMA) (Appendix A) [37]. The review is registered on PROSPERO (ID# CRD42023404861). We searched PubMed/MEDLINE, Embase, PsycINFO, Cumulative Index to Nursing and Allied Health Literature, and Cochrane Central databases on January 26, 2023, with the assistance of a research librarian. Our search was limited to studies published from 2013 onward. The search strategy, developed using synonyms for telemedicine and parents, is presented in Appendix B.

Using the Population, Intervention, Comparison Outcomes and Study (PICOS) [38] design as a guide, our inclusion criteria were as follows:

- Population: Parents included any family members providing a significant amount of childcare to support a child’s health and well-being. Children of these parents had to have a chronic or acute physical or mental health condition, or neurodevelopmental, intellectual or developmental disability. Chronic conditions were defined as lasting more than three months or occurred three times or more within one year, requiring ongoing medical attention or limiting activities of daily living [39]. Acute conditions were those with sudden onset, involving a short course of treatment (less than three months), and where a return to baseline was likely (e.g. acute bronchitis) [40].
- Intervention: Studies were included if they assessed the efficacy of parent-oriented mHealth interventions aimed at improving child or parental physical, psychological or developmental health. These interventions had to have been accessible through a mobile electronic device, including smartphones or tablets with interactive cellular communication capability [19, 29, 30]. Parents had to be among the users of the intervention to qualify for inclusion.
- Comparison: Control groups included usual care, no treatment, waitlist, or an active intervention.
- Outcomes: Parent outcomes included any observer or self-reported measure related to their ability to care for their child and their own psychological or physical health. Child health outcomes included any observer or self-reported measures related to the physical, psychosocial, or developmental health of the child.
- Study design: To be included studies had to be a randomized controlled trial of any size. We excluded dissertations, abstracts, and studies not published in English.

Perfect agreement on the application of eligibility criteria was achieved through two pilot tests involving 200 randomly selected abstracts, assessed by two independent coders (A.K. and I.Z.). After removing duplicates, all titles and abstracts, as well as full text articles, were screened in Covidence by two independent reviewers (A.K. and I.Z.). Any discrepancies were resolved by a third reviewer (P.P.).

### Data extraction procedures

A code book was developed by two authors (A.K. and I.Z.) to guide the extraction of information regarding the study, child, parent, and intervention. The information extracted about the intervention was adapted from the Template for Intervention Description and Replication checklist [41]. Data from a random sample of 10% of identified studies were extracted in duplicate by both authors (A.K. and I.Z.), achieving 100% agreement. Data from the remaining studies were extracted by one author (A.K.) and checked for accuracy by a secondary author (I.Z.) Any identified disagreements were resolved through discussion until full agreement was reached.

### Risk of bias

Using the Cochrane Risk-Of-Bias tool for randomized trials (ROB2) two reviewers (A.K. and E.M.) rated a random sample of 20% of studies in duplicate, achieving 80% agreement across all ROB2 domains [42]. The remaining studies were assessed individually, and any questions related to bias assessment were discussed as a group. Images for ROB2 were created using the Risk-of-bias VISualization tool [43].

### Outcomes and data synthesis

A narrative synthesis, tabulation, and descriptive analysis of the items extracted from the studies were conducted. To synthesize the cumulative impact of parent-oriented mHealth interventions, parent and child health outcomes were included in the narrative synthesis only if they were reported in two or more studies, with data on the remaining outcomes presented in tabular form.

The significant impact of the intervention on outcomes was determined based on a reported statistical difference of *p* < 0.05 between groups-over time, between groups, or a within a group from pre- to post-test. Data related to intervention content (type of intervention, frequency of use) and design features (co-design processes and theoretical frameworks utilized) were extracted and included in the narrative synthesis.

Interventions and parent and child health outcomes were organized using the framework developed by Van Houtven and colleagues (2011) for informal caregiver interventions [12]. This framework, designed for primary caregivers of adult patients, highlights that most caregiver-oriented interventions aim to improve or address four major categories pertaining to caregiving: (1) clinical knowledge, (2) psychological skills, (3) support seeking, and (4) quantity of caregiving (i.e. number of caregiving hours per week) [12]. Interventions were categorized according to this framework. Further, as outlined by the framework, parent and child health outcomes were classified as: (1) psychological health, (2) physical health, (3) healthcare utilization, and (4) economic status (i.e., changes in costs of health care services) [12].

Psychological health is defined as a dynamic state of internal equilibrium that enables individuals to use their abilities in harmony with the universal values of society, encompassing basic cognitive and social skills, as well as the ability to cope and function in social roles [44]. Parent psychological outcomes were further categorized as non-social, social and caregiving related, while child psychological outcomes were delineated into self-management related and non-social outcomes [12].

## Results

**Fig. 1:**
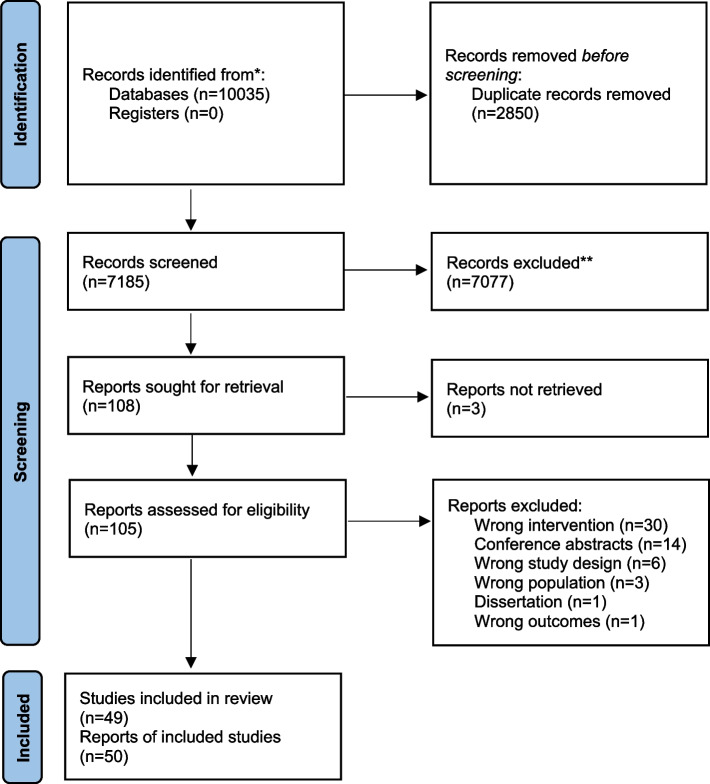
PRISMA flowchart of studies. This flowchart shows the number of records identified from the search (10,035), the number of records excluded based on title and abstract (7077), and the number of studies excluded based on the full article review (55), and the reason for exclusions. Fifty research articles (49 studies) were included in this analysis

Our search identified 10,035 titles and abstracts. After excluding 2850 duplicates, 7185 titles and abstracts were screened, and 108 full articles assessed for eligibility. Following screening, 50 articles pertaining to 49 unique studies were included (PRISMA diagram in Fig. 1).

### Characteristics and participant traits

**Fig. 2:**
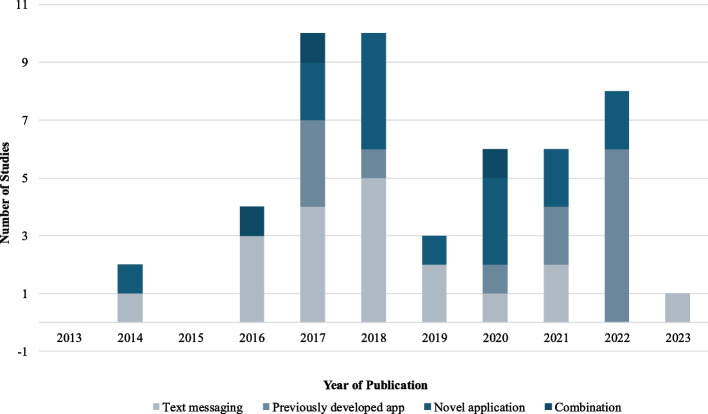
Number of studies and intervention type per year: This bar graph shows the number of studies (y-axis) published per year (x-axis). Each bar is broken down and colour coded according to intervention type

Table 1 summarizes the characteristics of the studies included in this research. The studies were published between 2014 and 2023, across 13 different countries, with the highest number published in 2017 (10 studies, 20%), 2018 (10 studies, 20%), and 2022 (8 studies, 16%) (see Fig. 2). The majority of the studies originated from the United States (26 studies, 53%) and China (8 studies, 16%).

**Table 1:** Study and participant demographics

				**Participant Ages**		**Participant Sex/Gender**		**Participant Race/Ethnicity**		
**Author, Year**	**Child Diagnosis**	**Location**	**Total Sample Size**	**Child Age**	**Parent Age**	**Child Sex/Gender**	**Parent Sex/Gender**	**Child Ethnicity/Race**	**Parent Ethnicity/Race**	**Parent Education**
Bernier et al., 2018 [45]	Type 1 Diabetes	United States	16 dyads (Intervention *n* = 8, Control *n* = 8)	Mean age in years (SD): 10.75 (3.44)	Not provided	Female: *n* = 10/16 (63%) Male: *n* = 6/16 (38%)	Not provided	White: *n* = 13/16 (87%), African American/Mixed: *n* = 3/16 (19%) Hispanic/Latino: *n* = 3/16 (19%)	Not provided	High school or lower *n* = 9/16 (56%), associate degree or higher *n* = 7/16 (44%)
Bhatia et al., 2020 [46]	Acute Lymphoblastic Leukemia	United States	444 children (Intervention *n* = 230, Control, *n* = 214)	Median age in years: 8.1 (IQR: 5.3–14.3 years)	Not provided	Males: *n* = 302/444 (68%) Females: *n*=142/444 (32%)	Not provided	Non-Hispanic White: *n* = 180/444 (40.5%) Hispanic: *n* = 170/444 (38.3%) African American: *n* = 43/444 (9.7%) Asian of Mixed: *n* = 51/444 (11.5%)	Not provided	Paternal education less than or equal to high schooln *n*= 195/444 (44%)
Castensøe-Seidenfaden et al., 2018 [47]	Type 1 Diabetes	Denmark	200 total (151 young people, 49 parents) Parents: Intervention *n* = 49, Control *n* = 0. Child: Intervention *n* = 76, Control group *n* = 75	Mean age in years (SD): 17.6 (2.6)	Not provided	Female: *n* = 81/151 (54%)	Not provided	Not provided	Not provided	Not provided
Cheung et al., 2022 [48]	Cancer	China	161 dyads (Intervention *n* = 81, Control *n *= 81)	Mean age in years (SD): 12.4 (2.4)	Mean age in years (SD): 42.8 (6.2)	Female: *n* = 68/161 (42.2%)	Female: *n* = 115/161 (71.4%)	Not provided	Not provided	Primary school or below: *n* = 7/161 (4.3%) Lower secondary school: *n* = 39/161 (24%) Upper secondary school: *n* = 76/161(47%) Tertiary education: *n* = 39/161(24%)
Coker et al., 2023 [49]	Asthma	United States	221 dyads (Intervention *n* = 111, Control: *n* = 110)	Mean age in years (SD): 5.8 (2.7)	Not provided	Female: *n* = 81/221 (27%)	Female: *n* = 194/221 (88%)	Not provided	Latino: *n* = 71/221 (32%) Non-Latino black: *n* = 42/221 (19%) Multiracial: *n* = 14/221 (6%) Non-Latino white: *n* = 60/221 (27%) Other: 26/211 (12%)	Less than high school: *n* = 32/221 (15%) High school or general educational development: *n* = 62/221 (28%), Some college or technical/vocational training: *n* = 79/221 (36%) College or more: *n* = 48/221 (22%)
Cooper et al., 2020 [50]	Congenital Heart Disease	United States	219 dyads (Intervention *n* = 109, Control *n* = 110)	Mean gestational age at birth in weeks (SD): Intervention: 38.9 (0.1) Control:38.8 (0.1)	Mean age in years (SD): Intervention: 30 (0.5) Control: 30 (0.6)	Female: *n* = 97/219 (44.3%) Male: *n* = 122/219 (55.7%)	Female: *n* = 213/219 (97.3%) Male: *n* = 6/219 (2.7%)	White: *n* = 173/219 (79%) Black: *n* = 28/219 (12.8%) Asian: *n* = 3/219 (1.4%) Mixed: *n* = 8/219 (3.7%) Other: *n* = 2/219 (0.9%) Unknown or not reported: *n* = 5/219 (2.3%)	Not provided	College: n = 120/219 (54.8%) Less than college: n = 73/219 (33.3%) Not reported or unknown: n = 30/219 (13.7%)
Everhart et al., 2017 [51]	Asthma	United States	28 dyads (Intervention *n* = 15, Control *n* = 13)	Mean age in years (SD): 9.67 (1.50)	Mean age in years (SD): 39.96 years (7.93)	Male: *n* = 19/28 (68%)	Female: *n* = 25/28 (89%)	Not provided	African American: *n* = 25/28 (89%)	Not provided
Fedele et al., 2021 [52]	Asthma	United States	33 dyads (Intervention *n* = 17, Control *n* = 16)	Mean age in years (SD): 13.18 (1.16)	Mean age in years (SD): 43 (9.31)	Female: *n* = 15/33 (45.5%)	Not provided	Caucasian/white: *n* = 5/33 (15.2%) Black/African American: *n* = 21/33 (63.6%) Latino: *n* = 3/33 (9.1%) Mixed or multiracial: *n* = 3/33 (9.1%) Other: *n* = 1/33 (3.0%)	Not provided	Not provided
Green et al.,2017; Smaldone et al., 2018 [53, 54]	Sickle Cell	United States	28 dyads (Intervention *n* = 18, Control *n* = 10)	Mean age in years (SD): 14.3 (2.6)	Not provided	Female: *n* = 12/28 (43%)	Female: *n* = 25/28 (89%)	Black: *n* = 14/28 (50%) Other: *n* = 13/28 (46%) Latino: *n*= 14/28 (50%)	Latino: *n *= 14/28 (50%)	High school or less: *n* = 16/28 (57%) Some college or graduate: *n* = 7/28 (25%)
Hajiabolhasani-Nargani et al., 2016 [55]	Autism Spectrum Disorder	Iran	64 dyads (Intervention *n* = 32, Control *n* = 32)	Mean age in years: 8	Mean age in years: 36	Male: *n* = 51/64 (80%)	Female: *n* = 64/64 (100%)	Not provided	Not provided	Bachelor's degree: *n* = 27/64 (42%)
Hannon et al., 2018 [56]	Type 1 Diabetes	United States	97 adolescents (Intervention *n* = 33/97, Control *n* = 33/97, Combined approach *n* = 31/97)	Average age in years (SD): Intervention = 14.5 (1.7) Control: 14.7 (1.5) Combined approach: 14.7 (2.0)	Not provided	Female: *n* = 48/97 (49%) Male: *n* = 49/97 (51%)	Not provided	Asian: *n* = 1/97 (1%) Black: *n* = 8/97 (8.2%) White: *n* = 80/97 (82.5%) More than one race: *n* = 3/97 (3.1%) Hispanic or Latino: *n* = 5/97 (5.2%)	Not provided	Not provided
Hemdi et al., 2017 [57]	Autism Spectrum Disorder	Saudi Arabia	62 dyads (Intervention *n* = 32, Control n = 30)	Average age in months (SD): Intervention: 63.18 (13.68) Control: 58.73 (14.07)	Mean age in years (SD): Intervention: 32.90 years (7.26 SD) Control: 34.43 years (6.65)	Not provided	Female: *n* = 62/62 (100%)	Not provided	Not provided	Less than high school: *n* = 10/62 (16%) High school: *n* = 20/62 (32%) bachelor's degree: *n* = 32/62 (52%)
Hilliard et al., 2020 [58]	Type 1 Diabetes	United States	80 dyads (Intervention *n* = 55, Control *n* = 25)	Average age in years (SD): 15.3 (1.5)	Not provided	Female: *n* = 47/80 (59%) Male: *n* = 33/80 (41%)	Female: *n* = 64/78 (80%)	Non-Hispanic white: *n* = 49/80 (61%) Non-Hispanic black: *n* = 10/80 (13%) Hispanic: *n* = 15/80 (19%) Other or more than one: *n* = 6/80 (7%)	Not provided	Not provided
Hinton et al., 2017 [59]	Disabilities	Australia	98 dyads (Intervention *n* = 51, Control *n* = 48)	Average age in years (SD): 6.01 (2.31)	Not provided	Male: *n* = 76/98 (78%) Female: *n* = 22/98 (22%)	Mother (biological or adoptive): *n* = 86/98 (88%) Stepmother: *n* = 2/98 (2%) Foster Mother: *n* = 1/98 (1%) Father (biological or adoptive) *n* = 6/98 (%) Grandmother: *n* = 3/98 (3%)	Not provided	Not provided	Not provided
Hofstetter et al., 2017 [60]	Chronic medical conditions	United States	295 dyads (Intervention *n* = 154, Control *n* = 141)	Age 11–12 years: *n* = 66/295 (22.4%) Age 13–17 years: *n* = 229/295 (77.6%)	Not provided	Male: *n* = 160/295 (54.2%) Female: *n* = 135/295 (45.8%)	Not provided	Latino: *n* = 239/295 (81.9%) Non-Latino black: *n* = 38/295 (13.0%) Non-Latino white: *n* = 5/295 (1.7%) Other/multiracial: *n* = 10/295 (3.4%)	Not provided	Not provided
Holtz et al., 2022 [61]	Type 1 Diabetes	United States	33 families (Intervention *n* = 23, Control group *n* = 10)	10 years old: *n* = 6/33 (18%) 11 years old: *n* = 4/33 (12%) 12 years old: *n* = 7/33 (215) 13 years old: *n* = 8/33 (24%) 14 years old: *n* = 4/33 (12%) 15 years old: *n* = 4/33 (12%)	25–34 years: *n* = 4/33 (12.1%) 25–44 years: *n* = 20/33 (60.6%) 45–54 years: *n* = 9/33 (27.3%)	Female: *n* = 20/33 (61%) Male: *n* = 13/33 (39%	Not provided	White: *n* = 30/33 (91%) Black or African American: *n* = 1/33 (3%) Hispanic or Latino: *n* = 2/33 (6%)	Not provided	Not provided
Huang et al., 2022 [62]	Type 1 Diabetes	China	92 dyads (Intervention *n* = 46, Control *n* = 46)	Mean age in years (SD): Intervention: 8.6 (3.2) Control: 8.3 (3.6)	Mean age in years: 30.75	Boy: *n* = 49/92 (53.3%) Girl: *n* = 43/92 (46.7%)	Not provided	Not provided	Not provided	Under high school: *n* = 18/92 (19.6%) High school: *n* = 27/92 (29.3%) Junior College: *n* = 31/92 (33.7%) bachelor's degree or higher: *n* = 16/92 (17.3%)
Jamali et al., 2022 [63]	Autism Spectrum Disorder	Iran	43 dyads (Intervention *n* = 21, Control *n* = 22)	Mean age in years (SD): Intervention: 8.18 (2.32) Control: 8.48 (2.84)	Mean age in years (SD: Intervention: 36.10 (5.13) Control: 38.86 (5.57)	Males: *n* = 33/43 (77%) Females: *n* = 10/43 (23%)	Intervention group: Mother: *n* = 20/21 (95%) Father: *n* = 1/21(5%) (Not provided for control group)	Not provided	Not provided	Primary: *n* = 6/43 (14%) Finished secondary: *n* = 14/43 (33%) associate or bachelor's degree: *n* = 20/43 (46.5%) master's degree: *n* = 3/43 (7%)
Jaser et al., 2019 [64]	Type 1 Diabetes	United States	120 adolescents (Phone group *n* = 30, Text *n* = 30, Control *n* = 60)	Mean age in years (SD): 14.83 (1.44)	Not provided	Male: *n* = 57/120 (47%) Female: *n* = 63/120 (53%)	Not provided	White, Non-Hispanic: *n* = 105/120 (88%) Other: *n* = 14/120 (12%) Unknown: *n* = 1/120 (0%)	Not provided	Not provided
Johansson et al., 2020 [65]	Obesity	Sweden	28 children (Intervention *n* = 15, Control *n* = 13)	Mean age in years (SD): Intervention: 8.4 (1.9) Control: 9.8 (2.2)	Not provided	Girl: *n* = 15/28 (53.4%) Boy: *n* = 13/28 (46.4%)	Not provided	Non-Nordic origin: *n* = 13/28 (46%) Nordic origin: *n* = 15/28 (53.6%)	Not provided	Not provided
Kenyon et al., 2019 [66]	Asthma	United States	41 children (Intervention *n* = 21, Control *n* = 20)	Mean age in years (SD): 5.9 (2.1)	Mean age in years (SD): 34.6 (10.0)	Male: *n* = 22/41 (54%) Female: *n* = 19/41 (46%)	Not provided	Black: *n* = 35/41 (85%) Other: *n* = 6/41 (15%)	Not provided	High school or less: *n* = 21/41 (51%) Some college and above: *n* = 20/41 (49%)
Khaksar et al., 2022 [67]	Pediatric hospitalizations	Iran	40 parents (Intervention *n* = 20, Control *n* = 20)	Not provided	20–24 years: *n* = 6/40 (15%) 25–29 years: *n* = 14/40 (35%) 30–34 years: *n* = 16/40 (40% 35–39 years: *n* = 2/40 (5%) 40–44 years: *n* = 2/40 (5%)	Not provided	Female: *n* = 40/40 (100%)	Not provided	Not provided	Under diploma: *n* = 6/40 (15%) Diploma: *n* = 18/40 (45%) Bachelor and higher: 16/40 (40%)
Kim et al., 2016 [68]	Obesity	South Korea	42 triads (children and both parents) (Intervention *n* = 23, Control *n* = 19	Mean age in years (SD): Intervention: 9.70 (1.49), Control 9.79 (1.62)	Mean age for in years (SD): Mothers: 40.78 (4.64), Fathers: 43 (5.81)	Male: 24/42 (57%) Female: 18/42 (43%)	Female: *n* = 42/84 (50%). Male: *n* = 42/84 (50%)	Not provided	Not provided	Mothers: < High school: *n* = 22/42 (52%) > College: *n* = 19/42 (45%) Fathers: < High school: *n* = 16/41 (39%) > College: *n* = 25/41 (61%)
Lee et al., 2017 [69]	Intellectual Disability and Obesity	Hong Kong (China)	115 families (Intervention *n* = 63, Control *n* = 52)	Mean age in years (SD): Intervention: 13.4 (2.7) Control: 15.3 (3.4)	Not provided	Male: *n* = 82/115 (71.3%) Female: *n* = 33/115 (28.7%)	Not reported	Not provided	Not provided	Paternal Education: Illiterate: *n* = 1/97 (1%) Primary Secondary: *n* = 19/97 (19.6%) Lower Secondary: *n* = 26/97 (27%) Upper Secondary: *n* = 34/97 (35%) Post-Secondary: *n* = 18/97 (18.6%) Maternal education Total: Primary School: *n* = 23/96 (24%) Lower Secondary: *n* = 29/96 (30%) Upper Secondary: *n* = 34/96 (35.4%) Post-Secondary: *n* = 10/96 (10%)
Lepley et al., 2020 [70]	Acute Illness/Pediatric Emergency Department	United States	98 dyads (Book *n* = 24, Book App *n* = 24, App n = 25, Control *n* = 25)	Median age in years: 2.0 (Range = 1.5–5 years)	Median age in years: 28 (IQR: 25–31.5 years)	Not provided	Female: *n* = 86/98 (87.8%) Male: *n* = 12/98 (12.2%%) Book Ap: Female: *n* = 21/24 (87.5%) Male: *n* = 3/24 (12.5%)	Not provided	Black: *n* = 45/98 (46%) Hispanic: *n* = 19/98 (19.4%) White: *n* = 27/98 (27.6%) Other: *n* = 6/98 (6%) Missing data *n* = 1	Less than high school: *n* = 11/98 (11.2%) Graduated high school: *n* = 33/98 (33.7%) Some college: *n* = 36/98 (36.7%) College degree: *n* = 18/98 (18.4%)
Liu et al., 2018 [71]	Herniorrhaphy surgery	China	418 dyads (WeChat *n* = 209, Leaflet *n* = 209)	Mean age range of 2.50–2.69 years	Age provided in ranges < 25 or 25: *n* = 37/418 (8.9%) 26–30: *n* = 242/418 (57.9%) 31–35: *n* = 116/418 (27.8%) 36–40: *n* = 21/418 (5%) > 40 or 40: *n* = 2/418 (0.48%)	Female: *n* = 111/418 (26.6%) Male: *n* = 307/418 (73.4%)	Female: *n* = 209/418 (50%) Male: *n* = 209/418 (50%)	Not provided	Not provided	Under high school: *n* = 32/418 (7.7%) High school: *n* = 152/418 (36.3%) Diploma: *n* = 112/418 (26.8%) bachelor's degree or higher: 122/418 (29.2%)
Luo et al., 2021 [72]	Cancer	China	103 dyads (Intervention *n* = 52 Control *n* = 51	Mean age in years (SD): Intervention: 5.48 (3.7) Control: 6.41 (3.6)	Mean age in years (SD): Intervention: 33.92 (5.4) Control: 33.22 (5.0)	Not provided	Female: *n* = 72/103 (70%) Male: *n* = 31/103 (30%)	Not provided	Not provided	Primary school: *n* = 8/103 (7.8%) High school: *n* = 66/103 (64%) College: *n* = 29/103 (28%)
McDuffie et al., 2018 [73]	Fragile X Syndrome	United States	20 dyads (Intervention *n* = 10, Control *n* = 10)	Mean age in years (SD): Intervention: 13.92 (2.26) Control: 12.46 (1.23)	Mean age in years (SD) only provided by groupings. Intervention group: 44.20 (6.00) Control group: 44 (6.13)	Male: *n* = 20/20 (100%)	Female: n = 20/20 (100%)	Not provided	Not provided	Mean years of education (SD). Intervention: 15.30 (1.77) Control: 15.40 (2.46)
Miloh et al., 2017 [74]	Inflammatory Bowel Disease	United States	51 children (Intervention *n* = 21 Control *n* = 30)	Not provided	Not provided	Not provided	Not provided	Not provided	Not provided	Not provided
Modi et al., 2016 [75]	Epilepsy	United States	25 adolescents and 10 parents (Adolescent text only: *n* = 5 Adolescent and parent text plus communication: *n* = 5 Adolescent application only: *n* = 5 Adolescent and parent application plus communication: *n* = 5 Epilepsy application for adolescents only: *n* = 5)	Mean age in years (SD): 15.7 (1.5)	Not provided	Female *n* = 12/25 (48%)	Not reported	Caucasian *n* = 23/25 (92%)	Not provided	Not provided
Moghimi et al., 2018 [76]	Mentally retarded children	Iran	70 parents (Intervention *n* = 35, Control *n* = 35)	Not provided	Mean age in years (SD): Intervention: 33.72 (3.8) Control: 34.1 (4)	Not provided	Female: *n* = 70/70 (100%)	Not provided	Not provided	Primary and secondary education: *n* = 25/70 (36%) High school: *n* = 30/70 (43%) Diploma and bachelor: *n* = 15/70 (21%)
Mruzek et al., 2019 [77]	Autism Spectrum Disorder	United States	32 children (Intervention *n* = 16, Control *n* = 16)	3 years: *n* = 21/32 (65.6%) 4 years: *n* = 4/32 (12.5%) 5 years: *n* = 5/32 (15.6%) 6 years: *n* = 2/32 (6.3%)	Not provided	Female: *n* = 6/32 (18.8%) Male: *n* = 26/32 (81.3%)	Not provided	Asian: *n* = 1/32 (3.1%) Black or African: *n* = 5/32 (15.6%) Caucasian/White: *n* = 22/32 (68.8%) Other/multiracial: *n* = 3/32 (9.4%)	Not provided	Not provided
Mulligan et al., 2022 [78]	Juvenile Idiopathic Arthritis	United States	203 families (Intervention *n* = 100, Control *n* = 103)	Mean age in years (SD): 6.1 (3.4)	Mean age in years (SD): 36.5 (6.5)	Female: *n* = 136/203 (67%) Male: *n* = 67/203(33%)	Female: *n* = 183/220 (83.2%) Male: *n* = 37/220 (16.8%)	Not provided	Not provided	</= GCSE or equivalent: *n* = 85/220 (38.7%) Advanced level of equivalent: *n* = 51/220 (23.2%) HNC or HND: *n* = 14/220 (6.4%) Degree or postgraduate: *n* = 70/220 (31.8%)
Nkoy et al., 2021 [79]	Medical Complexities	United States	50 dyads (Intervention *n* = 24, Control *n* = 26)	Mean age in years (SD): 8.5 (5.7)	Not provided	Male: *n* = 29/50 (58%) Female: *n* = 21/50 (42%)	Female: *n* = 43/50 (86%) Male: *n* = 4/50 (8%) Unknown: *n* = 3/50 (6%)	White: *n* = 38/50 (76%) Hispanic: *n* = 11/50 (22%) Other: *n* = 1/50 (2%)	White: *n* = 39/50 (78%) Hispanic: *n* = 7/50 (14%) Other: *n* = 4/50 (8%)	College incomplete: *n* = 22/50 (44%) College completed: *n* = 25/50 (50.5%) Unknown: *n* = 3/50 (65)
Phillips et al., 2014 [80]	Acute or Chronic tympanic membrane perforation	Australia	53 children (Intervention *n* = 30, Control *n* = 23)	Not provided	Not provided	Not provided	Not provided	All aboriginal children	Not provided	Not provided
Phipps et al., 2020 [81]	Cancer	United States	621 dyads (Intervention *n* = 310, Control *n* = 311)	Mean age in years (SD): Intervention: 8.3 (5.5) Control: 8.2 (5.5)	Mean age in years (SD): Intervention: 37.0 (8.6) Control: 36.7 (8.8)	Male: *n* = 345/621(55.6%) Female: *n* = 276/621 (44.4%)	Female: *n* = 549/621 (88.4%) Male: *n* = 72/621 (11.6%)	Not provided	White: *n* = 388/621(62.5%) Black: *n* = 77/621 (12.4%) Other/Unknown: *n* = 138/621 (22.2%)	Average highest grade completed (SD): Intervention: 13.6 (3.6) Control: 13.8 (3.5)
Salinero et al., 2021 [82]	Pediatric Emergency Room Visit	United States	123 dyads (Intervention *n* = 61, Control *n* = 62)	Mean age in years (95% CI). 2.2 (1.7–2.7)	Not provided	Male: *n* = 72/123 (59%) Female: *n* = 51/123 (41%)	Not provided	Not provided	Not provided	Not provided
Singer et al., 2018 [83]	Atopic Dermatitis	United States	30 dyads (Intervention *n* = 14, Control *n* = 16)	Average age in years (range) Intervention: 0.9 (0.3–2.2) Control: 1.4 (0.3–3.8)	Not provided	Female *n* = 17/30 (57%) Male: *n* = 13/30 (43%)	Not provided	Not provided	Not provided	Not provided
Swallow et al., 2014 [84]	Chronic Kidney Disease	United Kingdom	30 families (30 children and 41 parents) (Intervention: *n* = 14, control: *n* = 16)	Mean age in years (SD): Intervention: 9.1 (5.5) Control : 10.2 (5.7)	Mean age in years (SD): Intervention group: 42.7 (10.3) Control : 44.1 (8.3)	Female: *n* = 9/30 (30%) Male: *n* = 21/30 (70%)	Female: *n* = 22/41 (54%) Male: *n* = 19/41 (46%)	Not provided	White-European: *n* = 32/41 (78%) Afro-Caribbean: *n* = 1/41 (2.4%) South Asian: *n* = 8/41 (19.5%)	Not provided
Talisuna et al., 2017 [85]	Malaria	Kenya	1677 dyads (Intervention *n* = 828, Control *n* = 849)	Age ranges provided in months: < 12 years: *n* = 161/1677 (9.6%) 12–59 years: *n* = 1495/1677 (89.1%) 60 years: *n* = 21/1677 (1.3%)	Less than/equal to 20 years: *n* = 333/1677 (20%) 20–40 years: *n* = 1236/1677 (73.7%) > 40 years: *n* = 70/1677 (4.2%) Missing age: *n* = 38/1677 (2.3%)	Female: *n* = 787/1677 (46.9%) Male: *n* = 890/1677 (53%)	Female: *n* = 1589/1677 (94.8%) Male: *n* = 890/1677 (53%)	Not provided	Not provided	No formal education: 49/1677 (2.9%) Primary: *n* = 1096/1677 (65.4%) Secondary and above: *n* = 571/1677 (34%) Missing information: *n* = 7/1677 (0.42%)
Taveras et al., 2017 [86]	Obesity	United States	721 dyads (Intervention *n* = 360, Control *n* = 361)	Mean age in years (SD): 8.0 (3.0)	Mean age in years (SD): 38.4 (7.2)	Female: *n* = 368/721 (51%) Male: *n* = 353/721 (49%)	Not provided	Not provided	Not provided	< College graduate *n* = 356/721 (49.3%)
Teach et al., 2021 [87]	Asthma	United States	217 dyads (Intervention *n* = 107 Control *n* = 111)	Mean age in years (SD): 6.6 (2.3)	Mean age (SD): 33.8 (9.5)	Female: *n* = 85/217 (39.2%) Male: *n* = 132/217 (61%)	Female: *n* = 200/217 (92.2%) Male: *n* = 17/217 (7.8%)	Hispanic *n* = 1/217 (0.5%) African American *n* = 216/217 (99.5%)	Hispanic *n* = 1/217 (0.5%) African American *n* = 216/217 (99.5%)	Grade 1–11: *n* = 28/217 (12.9%) GED or 12 th Grade: *n* = 85/217 (39.2%) Some college/associate degree: *n* = 72/217 (33.2%) College Graduate: *n* = 32/217 (14.7%)
Weisman et al., 2018 [88]	Attention Deficit/Hyperactivity Disorder	Israel	39 children (Intervention *n* = 19, Control *n* = 20)	Mean age in years (SD): 9.56 (2.41)	Not provided	Female: *n* = 12/39 (31%) Male: *n* = 27/39 (69%)	Not provided	Not provided	Not provided	Not provided
Whitehouse et al., 2017 [89]	Autism Spectrum Disorder	Australia	75 families (Intervention n = 39, Control *n* = 36)	Mean age in months (SD): Intervention: 39.36 (8.50) Control: 40.25 (8.41)	Maternal mean age in years (SD): Intervention: 30.89 (4.63) Control: 32.25 (5.01) Paternal mean age in years (SD): Intervention: 34.06 (5.53) Control: 36.35 (5.58)	Not provided	Not provided	Not provided	Maternal Ethnicity (% Caucasian). *n* = 55/75 (73.3%) Paternal Ethnicity (% Caucasian) *n* = 55/75 (73.3%)	Maternal Education: Did not complete secondary school: *n* = 8/75 (11%) Secondary school completion: *n* = 10/75 (13%) Trade/technical certificate: *n* = 12/75 (16%) Undergraduate degree or higher: *n* = 42/75 (56%) Paternal Education. Did not complete secondary school: *n* = 10/75 (13%) Secondary school completion: *n* = 10/75 (13%) Trade/technical certificate: *n* = 17/75 (23%) Undergraduate degree or higher: *n* = 37/75 (49%)
Yang et al., 2016 [90]	Children undergoing tonsillectomies	Korea	61 dyads (Intervention n = 27, Control *n* = 34)	Mean age in years (SD): Intervention: 5.2 (1.3) control: 5.3 (1.3)	Mean age in years (SD): Intervention:35.8 (4) Control:36.3 (2.7)	Female: *n* = 18/61 (29.5%) Male: *n* = 43/61 (70.5%)	Female: *n* = 61/61 (100%)	Not provided	Not provided	</= High school: *n* = 18/61 (30%) College: *n* = 36/61 (59%) >/= College: *n* = 7/61 (11.5%)
Zhang et al., 2018 [91]	Type 1 Diabetes	United States	48 adolescents (Intervention *n* = 24, Control *n* = 24)	Mean age in years (SD): 14.7 (1.3)	Not provided	Female: *n* = 25/48 (52.1) Male: *n* = 23/48 (47.9)	Not provided	White, Non-Hispanic: 39/48 (81.2%) Other: *n* = 9/48 (18.8%)	Not provided	Not provided
Zhang et al., 2021 [92]	Congenital Heart Disease	China	84 dyads (Intervention *n* = 42, Control *n* = 42)	Mean age in months (SD): Intervention = 4.7 (4.3) Control = 4.9 (4.4)	Mean age in years (SD): Intervention = 29.1 (6.5) Control: 28.8 (7.1)	Not provided	Not provided	Not provided	Not provided	Under high school: *n* = 15/84 (17.9%) Highschool: *n* = 26/84 (31%) Junior college: *n* = 27/84 (32.1%) bachelor's degree or higher: 16/84 (19%)
Zhang et al., 2022a [93]	Congenital Heart Disease	China	84 dyads (Intervention *n* = 42, Control *n* = 42)	Mean age in months (SD: Intervention = 3.3 (3.1) Control = 3.6 (3.3)	Mean age in years (SD): Intervention: 28.5 (5.2) Control: 28.7(7.6)	Girls: *n* = 37/84 (44%) Boys: *n* = 47/84 (56%)	Not provided	Not provided	Not provided	Under high school: *n* = 13/84 (15.5%) High school: n = 34/84 (40.5%) Junior college: *n* = 27/84 (32%) bachelor's degree or higher: *n* = 10/84 (11.9%)
Zhang et al., 2022b [94]	Congenital Heart Disease	China	65 dyads (Intervention n = 35, Control *n* = 30)	Mean age in days (SD): Intervention = 18.0 (8.4) Control = 16.2 (7.3)	Age provided in ranges < 25 or 25 years: *n* = 7/65 (10.8%) 26–30 years: *n* = 19/65 (29.2%) 31–35 years: *n* = 23/65 (35.4%) 36–40 years: *n* = 16/65 (24.6%) > 40 or 40 years: *n* = 5/65 (7.7%)	Not provided	Not provided	Not provided	Not provided	Under high school: *n* = 10/65 (15.4%) High school: *n* = 23/65 (35.4%) Junior college: *n* = 24/65 (37%) Bachelor degree or higher: *n* = 13/65 (20%)

Out of the 49 studies, 19 (39%) were pilot or feasibility randomized controlled trials (RCTs), while 30 (61%) were full RCTs. The control groups used in these studies included usual care or wait-list control (22 studies, 45%) and active education interventions (27 studies, 55%). Studies in which usual care or waitlist control was used as a control group produced 27 statistically significant results (of 57 outcomes measured; 47%); while studies which used an active alternate intervention produced 23 of 55 (42%).

Sample sizes varied widely, ranging from 16 to 1,677 participants, with a mean of 155 and a standard deviation of 266. The most frequently studied child health condition was type 1 diabetes (8 studies, 16%), followed by autism spectrum disorder (ASD) (5 studies, 10%), asthma (5 studies, 10%), cancer (4 studies, 8%), obesity (4 studies, 8%), and heart disease (4 studies, 8%). Additionally, six studies (12%) focused on acute conditions, including post-operative management and recovery following hospital stays.

### Characteristics of parent-oriented interventions and their development

Table 2 outlines the characteristics of the interventions, the outcomes assessed, and the measurement tools used. The main objectives of these interventions were as follows:

- Improving or managing child health: 34 out of 49 studies (69%)
- Improving or managing parent health: 11 out of 49 studies (22%)
- Improving or managing both child and parent health: 4 out of 49 studies (8%)

**Table 2:** Interventional features and design

Author, Year	Intervention Target	Framework/Theoretical Model	Digital Modality	Intervention Description	Co-Design	Frequency of Use	Outcomes and Measurement Tool	
							**Outcome**	**Tool**
Bernier et al., 2018 [45]	Child health	Not discussed	Novel mobile application	An animation-based diabetes educational web application designed to run as an iBook	Each element and iteration of the intervention was reviewed with patients and their families in clinical and camp settings to guide the look and usability	Once	Parent Childhood diabetes knowledge	The Diabetes Knowledge Test [95]
Bhatia et al., 2020 [46]	Child health	Extended health belief model	Text messaging	Daily personalized text message reminder from an oncologist to the patient and parent to prompt direct supervised therapy	Not discussed	Daily for 16 weeks	Child Mercaptopurine adherence	Microelectronic technology used to record openings of bottles
Castensøe-Seidenfaden et al., 2018 [47]	Child health	Not discussed	Novel mobile application	mHealth app aimed at improving self-management of diabetes in young people and their parents	The app was developed using a participatory approach, but specifics not discussed	As needed for 12 months	Child HbA1 C	Bloodwork
							Child perceived competence managing diabetes	Perceived Competence in Diabetes Scale [96]
							Autonomy-supportive in providing general treatment	Health Care Climate Questionnaire [96]
							Child perceived burden of diabetes	Problem Areas in Diabetes Care Survey [97]
							Child severe hypoglycemic episodes	Self-report
							Child acute diabetes-related hospitalizations	Self-report
Cheung et al., 2022 [48]	Child health	Theoretical framework of the brief motivational interviewing	Previously developed mobile application	Mobile instant messaging-delivered brief motivational interviewing for parents to promote physical activity in pediatric cancer survivors	Not discussed	Weekly for 6 months	Child physical activity	Chinese University of Hong Kong Physical Activity Rating for Children and Youth Scale [98]
							Child fatigue	Chinese Version of the Fatigue Scale [99–101]
							Child left handgrip strength	Handheld dynamometer
							Child right handgrip strength	Handheld dynamometer
							Child peak expiratory flow rate	Peak flow meter
							Child Quality of Life	Chinese Version of the Pediatric QoL Inventory Scale [102]
Coker et al., 2023 [49]	Child health	Social cognitive theory	Text messaging	A parent-focused texting tool designed to provide parents with strategies for managing their child's asthma and improving communication with their child's primary care practitioner	Not discussed	2–3 times a week for 3 months	Parental Communication self-efficacy	Medical Competence Communication Scale [103]
							Parental asthma self-management knowledge	Asthma Self-Management Knowledge Questionnaire [104]
							Child health care utilization	Parent reported
							Asthma morbidity	Questionnaire validated previously in inner city asthma consortium study [105]
Cooper et al., 2020 [50]	Parental health	Not discussed	Previously developed mobile application	Retrieving data. Wait a few seconds and try to cut or copy again	Not discussed	Twice weekly for 4 months	Parent stress	Parenting Stress Index [106]
							Parent post-traumatic stress disorder	Post Traumatic Diagnostic Scale [107]
							Parent QoL	Ulm Quality of Life Inventory for Parents [108]
							Child weight	Medical records
							Child number of unplanned hospital readmissions	Medical records
Everhart et al., 2017 [51]	Parental health	Not discussed	Novel mobile application	A smartphone app for lower-income urban parents to improve management of their child's asthma	Not discussed	Daily for 14 days	Parent QoL	Pediatric Asthma Parent Quality of Life Questionnaire [109]
							Parent perceived stress	Perceived Stress Scale [110]
							Parent positive affect	Positive and Negative Affect Schedule [111]
Fedele et al., 2021 [52]	Child health	Pediatric self-management framework	Novel mobile application	A mobile app to improve asthma self-management in early adolescence	User-centered design methods used. Developed through iterative feedback from an advisory board composed of adolescent dyads	Twice a day for three weeks, as needed for 2 months	Parent family asthma management self-efficacy	Family Asthma Management System Scale [112]
							Parent family communication	Decision Making Involvement Scale [113]
							Child asthma control	Spirometer
							Child lung function	Forced expiratory volume
							Child QoL	Pediatric Asthma Quality of Life Questionnaire [109]
							Child self-efficacy	Asthma Management Efficacy Questionnaire [114]
Green et al.,2017; Smaldone et al., 2018 [53, 54]	Child health	Not discussed	Text messaging	Text-message reminders to improve hydroxyurea adherence	Partner community-based organization, Community League of the heights participated in designing the intervention and throughout the study	Daily for 3 months and as needed	Child fetal hemoglobin	Blood work
							Child ED visits	Parent reported
							Child Hospitalizations	Parent reported
							Child HU pharmacy refill	PDC calculation
							Child HU adherence	Morisky Self-Report Scale [115]
Hajiabolhasani-Nargani et al., 2016 [55]	Parental health	Not discussed	Text messaging	Mobile parenting skills education for mothers with autistic children	Not discussed	Daily for 2 months	Parent anxiety	Spielberger Questionnaire [116]
Hannon et al., 2018 [56]	Child health	Not discussed	Text messaging	Mobile self-monitoring blood glucose technology and family-centered goal setting	Not discussed	Daily for 6 months	Child HbA1 C number of SMBG tests	Blood work
							Child number of SMBG tests	SMBG data transmitted through telecare system
Hemdi et al., 2017 [57]	Parental health	Psychological models of stress and coping	Previously developed mobile application	Psychoeducation intervention delivered via WhatsApp for mothers of children with autism spectrum disorder	Not discussed	6 sessions (30 min each)	Parent stress, distress	Parenting Stress Index Short Form [106]
							Parent distress	Parenting Stress Index Short Form – Parent Distress Subscale [106]
							Parent anxiety	Hospital Anxiety and Depression Scale [117]
							Parent depression	Hospital Anxiety and Depression Scale [117]
							Parent happiness	Arabic Scale of Happiness [118]
							Parent parent child dysfunctional interaction	Parenting Stress Index Short Form [106]
							Child autism severity	Indian Scale for Autism Assessment [119]
							Child behaviour problems	Strength and Difficulties Questionnaire [120]
Hilliard et al., 2020 [58]	Child and parental health	Diabetes resilience model	Novel mobile application	A strengths-based mHealth app for parents of adolescents with Type 1 Diabetes	The app was designed with input from adolescents with T1D, their parents, and pediatric diabetes care providers	Daily for 3–4 months	Parent impact of diabetes	PedQL Family Impact Module and Diabetes Family Impact Scale [121]
							Parent family conflict	Diabetes Family Conflict Scale Revised [122]
							Parent perception of parents miscarried helping	Helping for Health Inventory [123]
							Parent parent-adolescent relationship	Parent-Youth Relationship Index [124]
							Parent distress	Problem Areas in Diabetes Measures for Parents [125]
							Child diabetes strengths	Diabetes Strengths and Resilience [126]
							Child QoL	Monitoring Individual Needs in Diabetes Youth Questionnaire [127]
							Child distress	Problem Areas in Diabetes Measures for Adolescent [128]
							Child degree to which diabetes bothered them	Six-point scale (no citation however internal reliability reported as high)
							Child self-management behaviours	Diabetes Self-Management Profile [129]
							Child self-care	Self-care Inventory Revised [130]
							Child HbA1 C	Blood work
Hinton et al., 2017 [59]	Child health	Self-regulatory framework for parents	Previously developed mobile application	A telehealth intervention aimed at treating and preventing severe behavioural, emotional, and developmental problems in children and adolescents with a disability	Parents were surveyed to determine what extra support they wanted included in the intervention	Weekly for 9 months	Parent satisfaction	The Client Satisfaction Questionnaire [131]
							Parent parenting practices and parenting self-efficacy	Child Adjustment and Parent Efficacy Scale – Developmental Disability [132]
							Parent adjustment	Parenting and Family Adjustment Scale [133]
							Child behavioural and emotional problems	Developmental Checklist – Primary Carer Version [134]
							Child prosocial behaviour and skills	Child Adjustment and Parent Efficacy Scale – Developmental Disability [132]
							Child total problems	Child Adjustment and Parent Efficacy Scale – Developmental Disability [132]
Hofstetter et al., 2017 [60]	Child health	Not discussed	Text messaging	Text message reminders for vaccination of adolescents with chronic medical conditions	Not discussed	Weekly for 5 months	Parent vaccine safety and effectiveness	Survey made for trial
							Child vaccines received	Immunization registry
							Child missed vaccine opportunities	Immunization registry
Holtz et al., 2022 [61]	Child health	Not discussed	Novel mobile application	App-based family communication intervention aimed to assist in adolescent self-management of Type 1 Diabetes	The team developed a paper prototype based on focus groups to determine the design, interest, design, and functionalities of the intervention. Additional focus groups were held and these sessions focused on what the adolescents wanted from the app including game functionalities and the ability to customize the app. They also wanted their parents to be engaged through the app. A prototype was developed and tested with 10 families who provided feedback	Daily for 12 weeks	Parent quality of family life	Impact on Family Scale [135]
							Child HbA1 C	Blood work
							Child self-management activities/adherence to diabetes medical regimens	Diabetes Behavior Rating Scale [136]
							Child QoL	Pediatric QoL Inventory Type 1 Diabetes Module [137]
Huang et al., 2022 [62]	Child and parental health	Not discussed	Previously developed mobile application	Telehealth education via WeChat to improve the quality of life of parents of children with Type- 1 Diabetes	Not discussed	Daily 6 months	Parent: anxiety	Self-rating Anxiety Scale [138]
							Parent depression	Self-rating Depression Scale [139]
							Parent QoL	World Health Organization Quality of Life Brief Scale [140]
							Child fasting blood glucose	Blood work
							Child hyperglycemia or hypoglycemia	Not reported
							Child HbA1 C	Blood work
							Child rehospitalizations	Not reported
Jamali et al., 2022 [63]	Child health	Not discussed	Previously developed mobile application	Occupation performance coaching for families of children with autism spectrum disorder by means of telerehabilitation	Not discussed	Twice a week for 8 weeks	Parent occupational performance	Canadian Occupational Performance Measure [141]
							Parent goal attainment	Goal Attainment Scale [142]
							Parent parenting self-efficacy	Child Adjustment and Parent Efficacy Scale—Developmental Disability [132]
							Child autism treatment evaluation	Autism Treatment Evaluation Checklist [143]
							Child total problems	Child Adjustment and Parent Efficacy Scale—Developmental Disability [132]
							Child prosocial behaviours	Child Adjustment and Parent Efficacy Scale—Developmental Disability [132]
							Child QoL	Short Form Health Survey [144]
Jaser et al., 2019 [64]	Child health	Not discussed	Text messaging	Positive psychology intervention for adolescents with Type 1 Diabetes	Not discussed	Weekly for 8 weeks	Child HbA1 C	Bloodwork
							Child perceived adherence to diabetes treatment	Self-Care Inventory [145]
							Child affect	Positive and Negative Affect Scale for Children [146]
							Child QoL	Pediatric QoL Inventory Type 1 Diabetes Module [147]
							Child coping with diabetes related stress	Responses to Stress Questionnaire [148]
							Child diabetes treatment recommendations	Average checks/day over the previous 30 days
Johansson et al., 2020 [65]	Child health	Not discussed	Combination of approaches	Mobile health support system for pediatric obesity treatment	Not discussed	Daily for 6 months	Child body mass index	Clinic visit
Kenyon et al., 2019 [66]	Child health	Not discussed	Text messaging	Text message reminders for controller adherence following hospital discharge in high-risk children with asthma	Not discussed	7 reminders over 30 days	Child inhaled corticosteroid adherence	Electronic sensors
							Asthma control	Child asthma control test (no reference provided)
Khaksar et al., 2022 [67]	Parental health	Family-centered care model	Previously developed mobile application	Bedside telehealth to improve family-centered care and decrease maternal stress in pediatric hospitalizations	Not discussed	Twice a day for 7 days	Parent stress	Stress response inventory [149]
Kim et al., 2016 [68]	Child health	Not discussed	Text messaging	Parent involvement intervention in developing weight management skills for both parents and overweight/obese children	Not discussed	Weekly for 5 weeks	Parent lifestyle	Lifestyle behaviour checklist [150]
							Parent child-parent relationship	Child-parent relationship scale [151]
							Child body mass index	Clinic visit
							Child dietary self-efficacy	Dietary self-efficacy scale [152]
Lee et al., 2017 [69]	Child health	Banduras social learning theory	Previously developed mobile application	School-based weight management program involving parents via mHealth for overweight and obese children and adolescents with intellectual disability	Experts (one physical activity specialist, one dietitian and one educational psychologist) and two school nurses were involved in the design	24 sessions over 6 months 1	Parent preferred cooking methods	Not reported
							Child QoL	Chinese version of the pediatric quality of life scale [153]
							Child self-esteem	Rosenberg’s self-esteem scale [154]
							Child self-efficacy in peer interaction	Children’s self-efficacy in peer interactions [155]
							Child perceived body shape	Perceived body shape scale [156]
							Child body image	Perceived body image questionnaire [157]
							Child nutrition self-efficacy	Nutrition self-efficacy scale [158]
							Child triceps skinfold thickness	Clinic visit
							Child BMI	Clinic visit
							Child subscapular skinfold thickness	Clinic visit
							Child waist to hip ratio	Clinic visit
							Child health knowledge and health behaviours	Scores on food pyramid tests, sports pyramid test, snack choice test (citation not provided)
							Child’s relationships with parents and teachers	Not reported
							Child body image perceptions	Self-figure rating scale [159]
Lepley et al., 2020 [70]	Child health	Not discussed	Novel mobile application	Acute illness educational intervention in the pediatric emergency department using mHealth or books	Not discussed	One time	Child non-urgent visits to ED	Parent reported and chart review
Liu et al., 2018 [71]	Child health	Not discussed	Previously developed mobile application	WeChat—assisted perioperative care instructions for parents of pediatric patients undergoing day surgery for herniorrhaphy	Not discussed	As needed for 7 days	Parent knowledge	10-item multiple choice knowledge questionnaire related to perioperative care (no citation given)
							Child rates of cancellation of surgery	Not reported
							Child postoperative complications	Not reported
							Child adverse events	Not reported
Luo et al., 2021 [72]	Parental health	Resilience framework	Previously developed mobile application	Mobile device-based resilience training program designed to reduce depressive symptoms and enhance resilience and quality of life in parents of children with cancer	Not discussed	Weekly for 8 weeks	Parent resilience	Connor Davidson resilience scale [160]
							Parent depression	Self-rating depression scale [139]
							Parent QoL	Short form of the 6-dimension health survey [161]
McDuffie et al., 2018 [73]	Child health	Not discussed	Novel mobile application	Distance delivery of a spoken language intervention for school-aged and adolescent boys with Fragile X Syndrome	Not discussed	Weekly for 12 weeks	Parent strategy use	Story-telling interaction examples (not citation provided)
							Child engagement	Story-telling interaction examples (not citation provided)
							Child story-related talking	Story-telling interaction examples (not citation provided)
							Child number of words	Story-telling interaction examples (not citation provided)
							Child mean length of utterance	Story-telling interaction examples (not citation provided)
Miloh et al., 2017 [74]	Child health	Not discussed	Text messaging	Text messaging to promote adherence in children with Inflammatory Bowel Disease	Not discussed	Patient's preference for 12 months	Child adherence	Morisky adherence questionnaire (citation not provided)
							Child disease activity	Pediatric Crohn’s/Ulcerative Colitis disease activity index (citation not provided)
							Child surgery	Chart review
							Child numbers of admission	Chart review
							Child ER visits	Chart review
							Child number of clinic no shows	Chart review
							Child weight change	Chart review
							Child height change	Chart review
							Child BMI change	Chart review
Modi et al., 2016 [75]	Child health	Not discussed	Combination of approaches	Text messaging and application-based adherence interventions in adolescents with epilepsy	Not discussed	Daily for 30 days	Child adherence over time	Medication event monitoring system
Moghimi et al., 2018 [76]	Parental health	Not discussed	Text messaging	Resilience teaching via short message service on stress of mothers of educable mentally retarded children	Not discussed	Four texts daily for 1.5 months	Parent resilience	Connor-Davidson resilience scale [160]
							Parent stress	Abidin parenting stress index [106]
Mruzek et al., 2019 [77]	Child health	Not discussed	Novel mobile application	iOS-based app for toilet training children with autism spectrum disorder	Not discussed	Several times a day for 12 weeks	Parent training fidelity	Parent training and treatment fidelity checklist (created for study)
							Parent anticipated of effectiveness	Parent expectancies scales [162]
							Parent satisfaction	Parent satisfaction survey (created for study)
							Child number of times the child urinated on the toilet	Parent reported
							Child number of toileting accidents	Parent reported
							Child's level of toileting independence	Parent reported
Mulligan et al., 2022 [78]	Parental health	Not discussed	Novel mobile application	Web-based tool for parents of children with Juvenile Idiopathic Arthritis	A focus group was conducted with 6 parents to ask their views on what the website should include. 2 focus groups with 12 health care professionals- 6 (50%) rheumatologists, 5 (42%) rheumatology nurse specialists, and 1 (8%) clinical psychologist-to ask their views on what the website should include. The resulting prototype website was tested by 7 parents and eight health professionals (4, 50%, rheumatologists; 2, 25%, rheumatology nurse specialists; 1, 13%, physiotherapist; and 1, 13%, clinical psychologist) to evaluate usability, navigation, structure, layout, and content. Minor changes were made to the website after this assessment	As needed for 12 months	Parent stress	Pediatric inventory for parents [163]
							Parent anxiety	Hospital anxiety and depression scale [117]
							Parent depression	Hospital anxiety and depression scale [117]
							Parent confidence in managing their child's arthritis	Parent’s arthritis self-efficacy scale [164]
							Parent effectiveness in managing their child's health care	Effective consumer adapted [165]
							Parent satisfaction with health care	Client satisfaction questionnaire [166]
							Child health related QoL	Child health questionnaire [167]
Nkoy et al., 2021 [79]	Child health	Not discussed	Novel mobile application	Home-monitoring application for children with medical complexity	Team previously identified preferences and key functionalities that parents found important for a CMC home-monitoring app.53 The results were used to design MyChildCMC and test it for usability with parents	Daily for 3 months	Parent satisfaction	Adapted version of the client satisfaction questionnaire [168]
							Child QoL	Parent perceptions of quality of life [169]
							Child ED or hospital admission	Chart review
							Child days in hospital	Chart review
Phillips et al., 2014 [80]	Child health	Not discussed	Text messaging	Mobile phone multimedia messages and text messages for improving clinic attendance for Aboriginal children with chronic otitis media	The style, design and interpretation of messages were determined in consultation with local Indigenous teachers and interpreters. The decision to use MMS rather than text alone was based on local advice that MMS were a more interesting, novel and potentially a more appealing method of communication than texts and that the videos could be shared among families	7 messages (one every 5 days for 6 weeks)	Child clinic visits	Chart review
							Child healed perforation	Clinic visit
							Child middle ear discharge	Clinic visit
							Child ear perforations size	Clinic visit
Phipps et al., 2020 [81]	Parental health	Not discussed	Novel mobile application	Web-based administration of a problem-solving skills intervention for parents of children with cancer	Development followed a user-centered design process, which included a series of formative focus groups to obtain parent perspectives. An initial website prototype was then reviewed by representative users from the study sites. Feedback from this review was used by the developers, and program changes were updated in a stepwise incremental process	Weekly for 8 weeks	Parent problem-solving skills	The social problem-solving inventory [170]
							Parent total mood disturbance	Profile of mood states scale [171]
							Parent symptoms of depression	Patient health questionnaire [172]
							Parent posttraumatic stress	Impact of events scale revised [173]
Salinero et al., 2021 [82]	Child health	Not discussed	Text messaging	Text message reminders to increase follow-up compliance after discharge from a pediatric emergency department	Not discussed	One time text	Child number of patients who followed up with a primary care clinic after discharge	Telephone follow up and chart review
							Child mean number of days to follow up	Telephone follow up and chart review
							Child number of patients who followed up with either the primary care clinic or the ED	Telephone follow up and chart review
Singer et al., 2018 [83]	Child health	Not discussed	Text messaging	Texting atopic dermatitis patients to optimize learning and eczema area and severity index scores	This pilot study involved receiving participant feedback regarding study experiences for future iterations of texting intervention	Daily for 42 days	Parent knowledge	16 question multiple choice atopic dermatitis knowledge quiz (no citation provided)
							Child extent and severity of eczema	Eczema area severity index [174]
Swallow et al., 2014 [84]	Parental health	Banduras self-efficacy theory	Novel mobile application	An interactive health communication application (online parent empowerment model) for supporting parents managing childhood long-term conditions	The OPIS application was developed in collaboration with families and health professionals. Interviews with 32 parents, 26 parents and 12 professionals were interviewed and feasibility testing	As needed for 20 weeks	Parent health literacy fathers support for managing disease	The rapid estimate for adult literacy in medicine [175]
							Parent management abilities	Family management measure [176]
							Parent Empowerment	Family empowerment scale [177]
							Parent fathers support for managing disease	Dad’s active disease support scale [178]
Talisuna et al., 2017 [85]	Child health	Not discussed	Text messaging	Text-message reminders on paediatric malaria treatment adherence and their post-treatment return to health facilities in in Kenya	Content, timing, understanding, and distribution had undergone extensive pre-testing with community members, parents, and patients at four facilities within the same county but outside of the study area	11 messages over 29 days	Child adherence to complete medicine course and individual doses	Pill count and self-report
							Child return to clinic	Clinic visit
Taveras et al., 2017 [86]	Child health	Positive outlier approach	Combination of approaches	Enhanced primary care and family coaching to leverage clinical and community resources to improve obesity and family-centered outcomes	The intervention was built on strategies recommended by a diverse group of stakeholders representing parents, children, pediatricians, and community members (chart audit, focus groups with obese children)	Twice-weekly text messages or emails and bimonthly meetings for 1 year	Parent empowerment	Child weight management subscale of the parent resource empowerment scale [179]
							Parent satisfaction	Parents were asked about whether they were satisfied with their child’s health care (no citation provided)
							Child body mass index	Chart review
							Child QoL	Pediatric quality of life – parent proxy [179, 180]
Teach et al., 2021[87]	Child and parental health	Not discussed	Text messaging	An intervention to manage parent psychosocial stress to improve asthma outcomes	Multidimensional stakeholder engagement included election of patient-centered outcome measures, refinement of intervention content and format, and language framing the study in a culturally appropriate manner	Weekly for 3 months	Parent stress	Perceived stress scale [110]
							Parent depression	Center for epidemiologic studies short depression scale [181]
							Parent resilience/optimism	Revised life orientation test [182]
							Parent quality of life	Pediatric asthma parent quality of life questionnaire [183]
							Parent use of stress management activities	Telephone follow-up
							Parent use of mental health resources	Telephone follow-up
							Parent sleep loss	Telephone follow-up
							Parent changing plans	Telephone follow-up
							Child symptom free days	Parent-reported
							Child asthma morbidity	Inner City Asthma Consortium [184]
							Child depression and anxiety	PROMIS parent proxy tool [185]
							Child reported adherence to asthma medication	Not reported
							Child asthma control	Childhood asthma control test [186]
							Child unscheduled asthma related health care utilizations	Telephone follow up
Weisman et al., 2018 [88]	Child health	Not discussed	Novel mobile application	A smartphone application in improving medication adherence, among children with ADHD	Not discussed	Daily for 8 weeks	Child pill count	Parent-reported
							Child ADHD symptom severity	Clinician rating scale [187], ADHD-Rating scale [188], and clinical global impression [189]
Whitehouse et al., 2017 [89]	Child health	Not discussed	Novel mobile application	ASD intervention programme that is accessible on a touch-screen device and targets a wide range of developmental abilities and complements early behavioural intervention	The app's acceptability and feasibility were established in a series of case studies	Daily for 6 months	Child developmental skills related to ASD	The autism treatment evaluation checklist [190]
							Child developmental abilities	Mullen scales of early learning [191]
							Child adaptive behaviours	Vineland adaptive behaviour scales [192]
							Child language and social development	Communication and symbolic behaviour scales developmental profile parent questionnaire [193]
							Child repetitive and restricted behaviours and interests	Repetitive behaviour scale-revised, behaviour flexibility rating scale [194]
Yang et al., 2016 [90]	Child health	Not discussed	Text messaging	Smartphone-based tonsillectomy education	The content of text message was developed based on a literature review and an assessment of patients'knowledge by a committee of clinical experts. The committee comprised two ENT specialists, four nurses with more than 7 years'clinical experience in the ENT wards, and one professor of college of nursing	Daily for 10 days	Parent (Mothers) knowledge	Self-reported survey developed by ENT specialists and researchers for study (no citation)
							Child anxiety	Modified eight-item self-report instrument
							Child sick-role behaviour	9-item questionnaire with a 3-point Likert-scale asking how well their child implemented measures to relieve their symptoms at home [195]
Zhang et al., 2018 [91]	Child health	Not discussed	Text messaging	Text messaging interventions to improve adherence in adolescents with Type 1 Diabetes	Not discussed	Weekly for 8 weeks	Child adherence to Type 1 Diabetes treatment regimen	Self-care inventory [145]
							Child glycemic control	Blood work
							Child adherence to medication	Downloading glucometer data to obtain frequency of blood glucose monitoring
Zhang et al., 2021 [92]	Parental health	Not discussed	Previously developed mobile application	Health education and care guidance at home via WeChat	Not discussed	Daily for 1 month	Parent anxiety	Self-rated anxiety scale [138]
							Parent depression	Self-rated depression scale [139]
							Parent QoL	World health organization quality of life [140]
Zhang et al., 2022a [93]	Child health	Not discussed	Previously developed mobile application	Remote health education and feeding guidance via WeChat	Not discussed	Daily for 1 month	Parent care ability	Family parent task inventory [196]
							Child nutritional status	STRONGkids scoring scale [197]
							Child albumin	Blood work
							Child Pre-albumin	Blood work
							Child hemoglobin	Blood work
							Child weight	Clinic visit
Zhang et al., 2022b [94]	Child and parental health	Not discussed	Previously developed mobile application	WeChat-assisted health education and preoperative care improve the mental state of parents of children with ventricular septal defect	Not discussed	Daily for 14.2 months	Parent transient and enduring levels of anxiety	State Trait Anxiety Inventory [198]
							Child complications	Clinic visit
							Child pneumonia	Clinic visit
							Child growth retardation	Clinic visit
							Child heart failure	Clinic visit

In terms of technology, 15 studies (31%) evaluated new apps specifically designed for health management, while 18 studies (37%) utilized text messaging. Additionally, 13 studies (26%) employed existing apps like WhatsApp or WeChat, and 3 studies (6%) used a combination of methods. Participants engaged with the interventions at different frequencies: daily (16 studies; 33%), weekly (10 studies; 20%), 2–3 times a week (7 studies; 14%), or as needed (6 studies; 12%).

Twelve articles (24%) discussed a theoretical framework or model that informed the content or structure of the interventions. These included Bandura’s self-efficacy theory (2 studies; 17%), psychological models of stress and coping (1 study; 12%), and resilience-based frameworks (1 study; 12%).

Nineteen studies (39%) reported that interventions were co-designed with various stakeholders: parents (4 studies; 21%), parents and their children (3 studies; 16%), healthcare professionals (2 studies; 11%), and community members (2 studies; 11%). The most common co-design methods included establishing advisory boards with patients, parents, or community partners (4 studies; 21%), conducting focus groups with end-users to develop content for prototypes (3 studies; 16%), usability testing of prototypes (2 studies; 11%), and conducting surveys to assess the needs of parents and children (1 study; 5%).

### Interventional target

Thirty studies (61%) aimed to enhance parents'clinical skills and knowledge. Of these, 22 studies (45%) focused on improving parents'abilities to manage their child's condition, while 8 studies (16%) targeted increasing parents'understanding of the disease and its expected clinical course.

Seventeen studies (35%) aimed to boost parents'psychological skills, specifically in two areas: self-efficacy for performing tasks related to their child's care (6 studies) and coping strategies for managing the demands of caring for a child with a health condition (11 studies).

Additionally, three studies (6%) sought to provide social support to parents, but this support was secondary to the primary goals of improving clinical or psychological skills. Notably, no interventions aimed at reducing the overall caregiving burden, such as the number of tasks or hours spent providing care [12].

### Parent health outcomes

The parent health outcomes identified were exclusively related to psychological health. No studies examining physical health, economic factors, or healthcare utilization outcomes were identified (see Table 3).

**Table 3:**
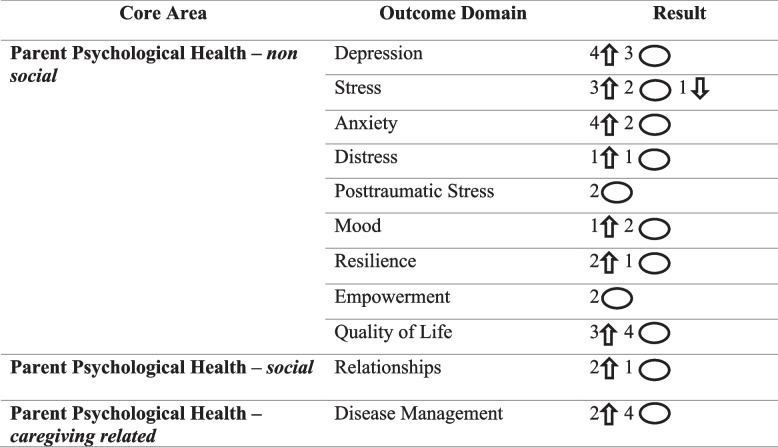
mHealth intervention impact on parental health outcomes

Upwards arrows indicate significantly positive (*p*< 0.05) patient impact in the mHealth group, circles indicate no significant effect noted, downwards arrows indicate a significantly negative (*p*< 0.05) patient impact in the mHealth group

### Parent psychological health

#### Non-social psychological outcomes

Depression scores showed significant improvement in four out of seven studies (57%) that assessed this outcome [57, 62, 72, 92], using a variety of validated tools. mHealth interventions significantly reduced stress compared to the control group in three out of six studies (50%) [57, 67, 76]. However, in one study (17%), the control group, which received an in-person version of the intervention, reported a significantly greater decrease in stress than the intervention group [87]. Parent anxiety scores were measured in six studies, and significant improvements related to the intervention were observed in four of these studies (67%) [55, 62, 92, 94].

Resilience showed significant improvement in two out of three studies (67%) that measured this construct [72, 76]. Quality of Life (QoL) was assessed in seven studies, with significant improvements observed in three of these studies (43%) [61, 62, 92].

### Social psychological outcomes

The quality of a parent's relationship with their family or ill child was measured in three studies. Of these, two studies (67%) reported significant improvements in the intervention group [59, 68]. These studies measured the outcome using a subscale of the Parenting and Family Adjustment Scale [133] and the child-parent relationship scale [151].

### Caregiving related psychological outcomes

Parents'ability to manage their child's condition was assessed six times across four studies, showing significant improvement from baseline in two of the six measurements (33%). Specifically, improvements were noted in parents'confidence in managing their child's arthritis [78] symptoms and their reported ability to manage their child’s chronic kidney disease [84].

Seventeen studies examined parents'satisfaction with the intervention, using various methods, including qualitative interviews (2 studies), the Client Satisfaction Questionnaire (3 studies) [131], and investigator-developed surveys (10 studies). All studies reported high satisfaction with the mHealth intervention, except for one study where only 37% of parents found the application somewhat useful, compared to 70% who endorsed the utility of the control treatment [70]. Additionally, one study reported no difference in satisfaction between the mHealth and control groups [77].

### Child health outcomes

Child health outcomes included physical health related to diabetes, cardiovascular issues, asthma, and neurodevelopmental disorders, as well as healthcare utilization, psychological health, and disease management (see Table 4).

**Table 4:**
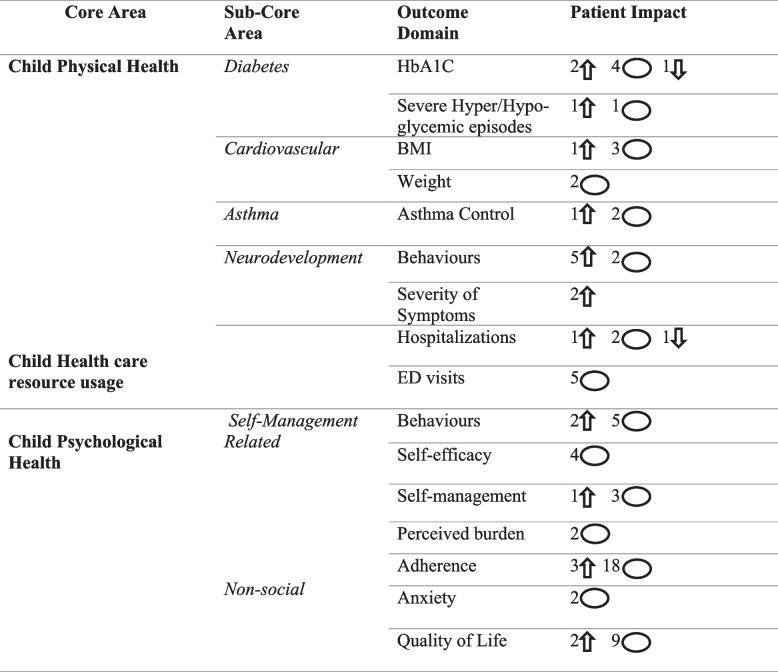
mHealth Intervention impact on child health outcomes

Upwards arrows indicate significantly positive (*p*< 0.05) patient impact in the mHealth group, circles indicate no significant effect noted, downwards arrows indicate a significantly negative (*p*< 0.05) patient impact in the mHealth group

### Child physical and neurodevelopmental health

#### Diabetes

Diabetes-related health was assessed in seven studies, focusing on glycemic control as measured by Hemoglobin A1C (HbA1 C) levels. Two studies (29%) reported significant improvements in glycemic control in the mHealth group [61, 62]. However, in one study (14%), the significant improvement favored the control group [47].

### Cardiovascular health

The most commonly assessed cardiovascular health outcomes were body mass index (BMI), evaluated in four studies, and weight, assessed in two studies. A significant improvement in BMI, favoring the use of mHealth interventions, was observed in one out of the four studies (25%) [65].

### Asthma

Asthma control was evaluated in three studies using either spirometry or the Child Asthma Control Test [186]. Significant improvement was observed in one of the studies (33%) [52].

### Neurodevelopmental disorders

The neurodevelopmental disorders identified in the studies included autism spectrum disorder (ASD) (3 studies), attention deficit hyperactivity disorder (ADHD) (1 study), and multiple neurodevelopmental disorders (1 study). Children's behaviors associated with these disorders were measured seven times across four studies, focusing on behavioral and emotional problems [59], pro-social behaviors [59, 63], and adaptive behaviors [89]. Of these seven assessments, significant improvements from baseline were observed in five (71%) instances [57, 59, 63].

The impact of mHealth interventions on the severity of ASD [57] and ADHD [88] symptoms was evaluated in one study each, with both studies reporting significant positive improvements.

### Healthcare resources

Healthcare resource usage was assessed in terms of hospitalizations (4 studies) and emergency department visits (5 studies). Among the studies that evaluated hospitalizations, one study (25%) found that hospitalizations were significantly lower in the intervention group [62]. In another study (25%), significantly fewer infants in the control group were readmitted to the hospital compared to those in the intervention group [50].

### Child psychological

#### Self-management related psychological outcomes

Children's ability or confidence in managing their health conditions was assessed across 17 outcomes in 9 studies. These included management behaviors (7 outcomes), self-efficacy (4 outcomes), self-management (4 outcomes), and perceived burden of disease-related problems (2 outcomes). Significant differences in self-management activities and health knowledge favoring the intervention group were noted in three instances across two studies (19%) [61, 69].

Adherence to treatment was evaluated in 21 instances across 14 studies, with significant improvements in the intervention group observed in three studies (14%) [60, 71, 74]. In one additional study, no significant difference was found overall; however, an analysis of intervention engagement revealed that participants who engaged more with the intervention showed significant improvements in medication adherence [91].

### Non-social psychological outcomes

Pediatric quality of life (QoL) was assessed eleven times across ten studies, focusing on either disease-specific QoL or generic health-related QoL. Of these measurements, significant improvements from baseline in the intervention group were observed in two instances (17%) [48, 69]. 

### Outcome effects of mhealth intervention features and design

Table 5 presents the outcome results based on the features and design modalities of mHealth interventions. In the 19 studies that reported co-designing interventions, a total of 58 parent and child health outcomes were assessed, with statistically significant improvements observed in 20 outcomes (34%).

**Table 5:**
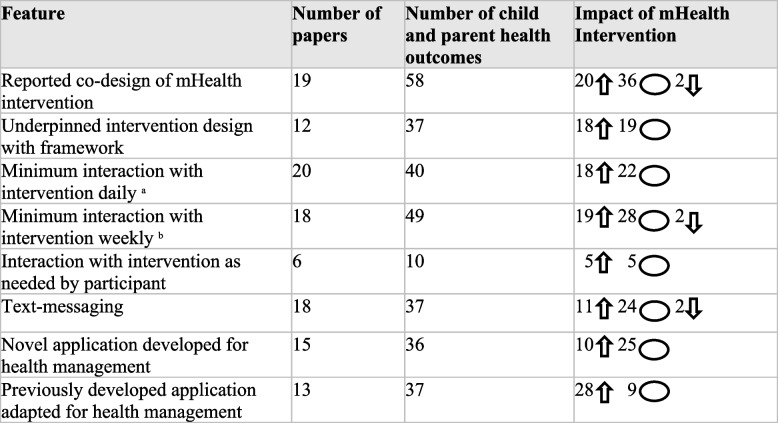
Trends towards significant differences per mHealth intervention feature and design

A= participant interaction with intervention includes daily, twice a day, several times a day and four times a day.

B= participant interaction with intervention includes weekly, 2-3 times a week and 7 reminders over 30 days.

Upwards arrows indicate significantly positive (*p*< 0.05) patient impact in the mHealth group, circles indicate no significant effect noted, downwards arrows indicate a significantly negative (*p*< 0.05) patient impact in the mHealth group

Among the 12 (24%) studies that employed a theory-driven intervention, 37 health outcomes for children and parents were measured, with 18 outcomes (49%) showing significant improvements in the parent-oriented mHealth group. In studies where participants were required to engage with the intervention at-least daily, 18 out of 40 assessed outcomes (45%) showed significant improvement. Similarly, in studies that required at least weekly (and less frequently than daily) interaction with the intervention, 19 out of 49 outcomes (39%) demonstrated significant improvement.

Text messaging and novel mobile applications developed by the research teams for health management significantly improved 30% (11 out of 37) of the assessed outcomes across 18 studies, and 28% (10 out of 36) across 15 studies, respectively. In contrast, previously developed applications that were adapted for health management showed significant improvements in 76% (28 out of 37) of parent and child outcomes across 13 studies.

### Risk of bias assessment

**Fig. 3:**
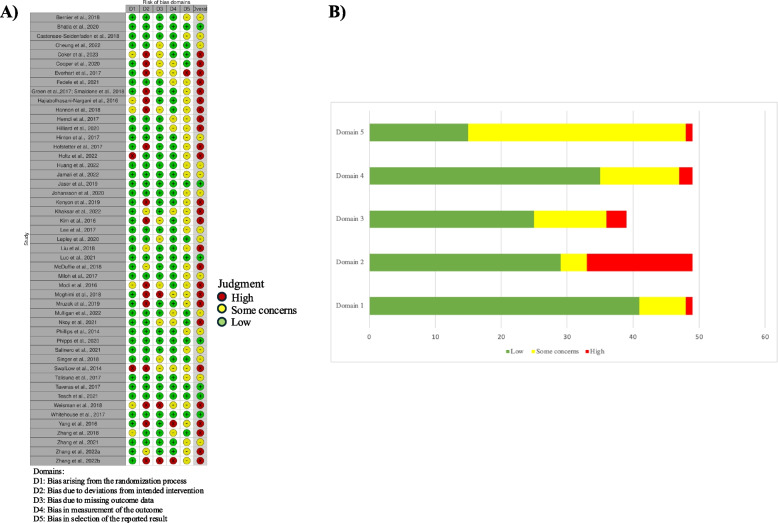
Risk of bias assessment. Individual bias assessment (**a**) and (**b**) overall summary for included randomized controlled trials assessed using the Cochrane collaboration tool

According to the Cochrane ROB2 tool, 26 studies (53%) were assessed as having a high risk of bias, 16 studies (33%) had some concerns, and 7 studies (14%) had low concerns (see Fig. 3a). The bias domain with the highest risk across the studies was deviations from the intended interventions, affecting 16 out of 49 studies (33%). In contrast, the domain with the lowest risk was the randomization process, which was properly implemented in 41 out of 49 studies (84%) (see Fig. 3b).

## Discussion

We synthesized the literature examining the effectiveness of parent-oriented mHealth interventions on parent and child health outcomes and identified key content and design features that may have contributed to their effectiveness. We identified 49 studies, most frequently published in developed countries, including the United States (26 studies), with more than half scoring high-risk for bias. Overall, the identified interventions were found to be highly acceptable to parents and improved parent psychological health, with the largest impact observed in non-social psychological outcomes including a 57% improvement in depression, a 67% improvement in anxiety, and a 67% improvement in resilience.

Early evidence also suggests the utility of these interventions in improving child health outcomes, particularly regarding neurodevelopmental health outcomes, in which measured outcomes showed a 71% significant improvement. Interventions frequently connected with participants used texting or novel applications daily or weekly but were infrequently underpinned by theoretical health behaviour frameworks or developed through end-user co-designed. Evaluated interventions that involved daily engagement (45% improvement) or weekly engagement (39%), used co-design development techniques (34%), were theory-driven (49%), and included mobile applications (76% for previously established and 28% for novel) demonstrated strong effectiveness in improving both parent and child health outcomes.

Our finding that parent-oriented mHealth interventions are more common and beneficial in the context of chronic childhood conditions compared to acute ones may reflect the prolonged and complex care required from parents in chronic situations [199]. Interventions applied in the context of neurodevelopmental disorders, including ASD, cardiovascular disease and cancer, had the largest impact on both parent and child health outcomes. Parents of children with these conditions often experience a significant subjective burden, characterized by intense physical, emotional, social, and financial stress, which is linked to the extensive hours and demanding care tasks these conditions require [200–202]. It is important to further explore whether these types of interventions increase the burden on parents and families of children with chronic conditions, as well as on the healthcare system, in terms of cost and time. While previous reviews have shown that these interventions are relatively low-cost [203], more research is needed in pediatrics to assess their cost-effectiveness compared to usual care. Additionally, further work is required to determine if mHealth interventions place additional strain and expectations on parents of sick children and on healthcare providers who are already overburdened [204, 205]. Interestingly, in some studies, control groups showed significantly better outcomes than the intervention groups. This highlights the need for careful adaptation of interventions when transitioning to mobile-based formats, as well as the need to consider the therapeutic benefit of integrating person-based care into digital technology design [206].

Interventions aimed at enhancing caregiving capacity and support may be particularly effective for these parents [200–202]. However, demographics information about the parents, aside from sex, was inconsistently reported, making it challenging to determine who would benefit most from these interventions. However, most parents identified as females, specifically mothers. The lack of engagement of fathers in parent-oriented interventions has been previously noted and is linked to beliefs about gender roles regarding caregiving, lack of relevant interventions, and limited awareness of available interventions [207].

Less than 20% of the identified interventions aimed to enhance parental psychological skills, despite evidence showing positive impacts on parental psychological health, including a 67% improvement in reported anxiety and a 57% improvement in depression. This lack of focus on the well-being of parents has been noted previously and should be a key research direction in the field [208–210]. Our review demonstrates that when mHealth applications do consider parent psychological health in their design, they positively impact both caregiver and child outcomes. Such interventions align with the family-centered care model integral to pediatrics and address an expressed need among parent caregivers of children with chronic conditions [200, 211].

Although some evaluated interventions resulted in significant positive child health outcomes, this was not consistent across all studies. Challenges related to collecting health-related subjective ratings from younger children may partly explain this discrepancy [212]. Many identified studies relied on parent proxy-reported outcomes, which may not accurately reflect the child health status, particularly concerning psychological health outcomes [213]. Additionally, longer intervention periods may be necessary to improve various child objective and functional health outcomes such as HbA1C or blood pressure, as well as to enhance parental caregiving self-efficacy, and, consequently, child health [214].

Interventions that incorporate co-design methodologies in their development (34% improvement), are theory-driven (49%), and include more frequent interaction with users (45%) appear to be effective. Our findings suggest that co-design practices appear useful for developing more widely utilized digital interventions for parents [215, 216]. Although this approach has been inconsistently applied, the importance of grounding intervention features and function in behavioral change frameworks or models to enhance impact has been previously demonstrated and is reflected in our findings [217]. However, while we report on whether or not studied interventions were based on a theoretical framework, we cannot comment on the extent to which interventions correctly applied the framework’s tenets in their design. Due to the complex and multi-component nature of mHealth interventions, comparisons between those that include these elements and those that do not are challenging [218]. Future research should focus on identifying the features that are most effective in different patient settings and age group—a goal that could be achieved through co-design efforts involving parents, pediatric patients, and clinicians.

Questions remain around the methods for successfully implementing parent–child mHealth interventions into clinical practice. In particular, the lack of digital inclusion—encompassing access to and the relevance of digital technologies for individuals or groups—limits many populations’ capacity to engage with and potentially benefit from these care models [219]. Ensuring that digital health interventions are designed equitably is critical to minimizing the digital healthcare divide. Frameworks such as the eHealth Literacy Framework may inform the design and implementation of digital interventions, improving their applicability across target populations [220]. Particular considerations, including those related to literacy and user experience norms, are essential when developing interventions for pediatric patients and their parents. One option to address this issue is to develop applications that include specific profiles for both parent and child users.

Other concerns include how data from these interventions can be effectively integrated into child electronic health records, particularly when outcomes are parent-proxy reported due to the child’s age or illness, or when the data pertain to parental health status rather than that of the child [208]. Additionally, given the family and treatment related demands, engaging parents of children with chronic conditions in consistent and longitudinal use of interventions poses a barrier to implementation [221, 222]. Engagement strategies such, as gamifying interventions and utilizing push notifications, have been suggested to improve retention in mHealth studies [223].

### Limitations

The studies identified in this review are not without limitations. Several studies exhibited a high risk-of-bias due to a lack of participant blinding. Although it is challenging to blind participants without an active control group, future studies could blind outcome assessors and analysts. Additionally, most studies were published in high income countries. Given the pressing need to increase access to high quality child healthcare in lower income countries, future research should focus on evaluating mHealth interventions in these regions.

This review also has limitations. Due to the wide variety of health conditions and types of interventions, a meta-analysis was not feasible. Furthermore, we only included RCTs, so we cannot comment on the results of mHealth evaluations using different study designs. In addition, to capture the full extent of mHealth interventions in the literature, we included pilot or feasibility studies that were not powered for statistical significance. Finally, studies not published in English were excluded, which may limit our understanding of these interventions in other cultural contexts.

## Conclusions

Overall, parent-oriented mHealth interventions appear to improve parent psychological health and may positively affect child health. Given these encouraging findings and the widespread accessibility of mobile digital devices, mHealth interventions could significantly enhance the quality of family-centered pediatric healthcare. Intervention functionalities and design features, such as co-design and the use of health behaviour theoretical frameworks, may be valuable in amplifying the impacts of developed mHealth applications. Further research is needed to elucidate when and how to apply these technologies most effectively within pediatric care. Taken together, parent-oriented mHealth interventions represent a promising tool for improving outcomes for both parents and their children, facilitating family-centered care.

## Supplementary Information

Supplementary Material 1.

Supplementary Material 2.

## Data Availability

No datasets were generated or analysed during the current study.

## Abbreviations

ADHD: Attention Deficit Hyperactivity Disorder
ASD: Autism Spectrum Disorder
HbA1 C: Hemoglobin A1C
mHealth: Mobile Health
PRISMA: Preferred Reporting Items for Systematic Reviews and Meta-Analyses
QoL: Quality of Life
RCT: Randomized Controlled Trial
ROB2: Risk-Of-Bias Tool for Randomized Trials
