# Supplementary material for: Understanding the effectiveness and design of parent-oriented mobile health interventions: a systematic review and narrative synthesis
Source: BMC Pediatr. 2025 May 10;25:372. doi: 10.1186/s12887-025-05656-y (PMC12065154; doi:10.1186/s12887-025-05656-y)
Supplement: Supplementary file 2 — Supplementary Material 2. [file 12887_2025_5656_MOESM2_ESM.docx]

**Appendix B Medline Search Strategy**

| **#** | **Searches** |
| --- | --- |
| 1 | Telemedicine/ |
| 2 | Mobile Applications/ |
| 3 | Cell phone/ or smartphone/ or text messaging/ |
| 4 | (telemedicine or tele-medicine or telehealth or tele-health).tw,kf. |
| 5 | (mHealth or m-Health or eHealth or e-Health).tw,kf. |
| 6 | ((mobile or digital or electronic) adj3 health).tw,kf. |
| 7 | ((smart or mobile or cell or wireless) adj3 phone*).tw,kf. |
| 8 | ((smart or mobile or cell or wireless or tablet or interactive) adj3 app*).tw,kf. |
| 9 | (text adj3 messag*).tw,kf. |
| 10 | (play adj3 technolog*).tw,kf. |
| 11 | 1 or 2 or 3 or 4 or 5 or 6 or 7 or 8 or 9 or 10 |
| 12 | Parents/ or Fathers/ or Mothers/ or Single Parent/ or Surrogate Mothers |
| 13 | Caregivers/ |
| 14 | Legal Guardians/ |
| 15 | (parent* or stepparent* or father* or stepfather* or mother* or stepmother* or guardian* or caregiver*).tw,kf. |
| 16 | 12 or 13 or 14 or 15 |
| 17 | 11 and 16 |
| 18 | randomized controlled trial [pt] |
| 19 | controlled clinical trial [pt] |
| 20 | randomized [tiab] |
| 21 | placebo [tiab] |
| 22 | drug therapy [sh] |
| 23 | randomly [tiab] |
| 24 | trial [tiab] |
| 25 | groups [tiab] |
| 26 | 18 or 19 or 20 or 21 or 22 or 23 or 24 or 25 |
| 27 | animals [mh] NOT humans [mh] |
| 28 | 26 not 27 |
| 29 | 17 and 28 |
